# Supplementary material for: Auto-amplification and spatial propagation of neutrophil extracellular traps
Source: Commun Biol. 2024 Mar 29;7:386. doi: 10.1038/s42003-024-06074-z (PMC10980821; doi:10.1038/s42003-024-06074-z)

Supporting information (SI)

Auto-amplification and Spatial Propagation of Neutrophil Extracellular Traps

Authors: Pan Deng[1,2], Alec Xu[1,2], Peter M. Grin[2,3], Kerryn Matthews[1,2], Simon P. Duffy[1,2,4], and Hongshen Ma[1,2,5,6]

Affiliations
[1]Department of Mechanical Engineering, University of British Columbia
[2]Centre for Blood Research, University of British Columbia
[3]Department of Biochemistry and Molecular Biology, University of British Columbia
[4]British Columbia Institute of Technology
[5]School of Biomedical Engineering, University of British Columbia
[6]Vancouver Prostate Centre, Vancouver General Hospital

Corresponding Author
Hongshen Ma
2054-6250 Applied Science Lane
Vancouver, BC, Canada V6T 1Z4
604.722.5382
hongma@mech.ubc.ca

Supplementary Figure 1: Percentage of NETs in dHL-60 cells treated with different stimuli in nanowells. Ionomycin (6 µM), lipopolysaccharide (LPS, 100 µg/mL), phorbol myristate acetate (PMA, 200 nM). For each group, the values shown are the average from 12 different 5×5 nanowell units across three individual microwells; Error bars represent standard deviation.

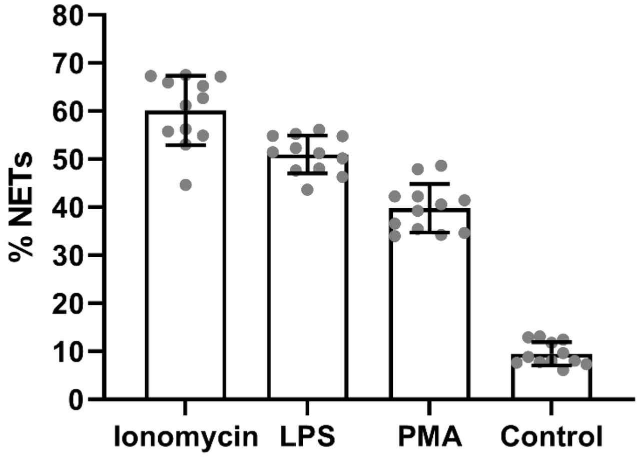

Supplementary Figure 2. Residual ionomycin was effectively removed after the washing steps (n=16 per group). For each group, the values shown are the average from 16 different 5×5 nanowell units across three individual microwells; Error bars represent standard deviation.

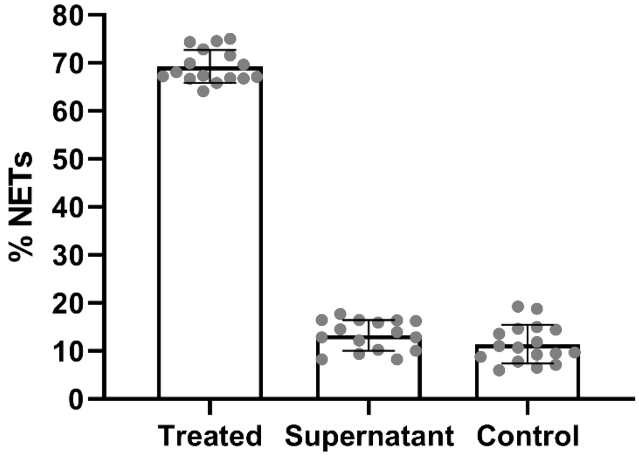

Supplementary Figure 3. NETosis propagation starting from a plaque of stimulated cells. (A) Representative images of NETosis propagation at different time points. The NETs plaque formed from stimulated cells (treated cells) was pre-stained with Hoechst (blue). The untreated cells releasing NETs are stained using the membrane-impermeable DNA stain SYTOX Green (green). (B) SYTOX Green images of untreated cells from the selected areas A1 and A2.

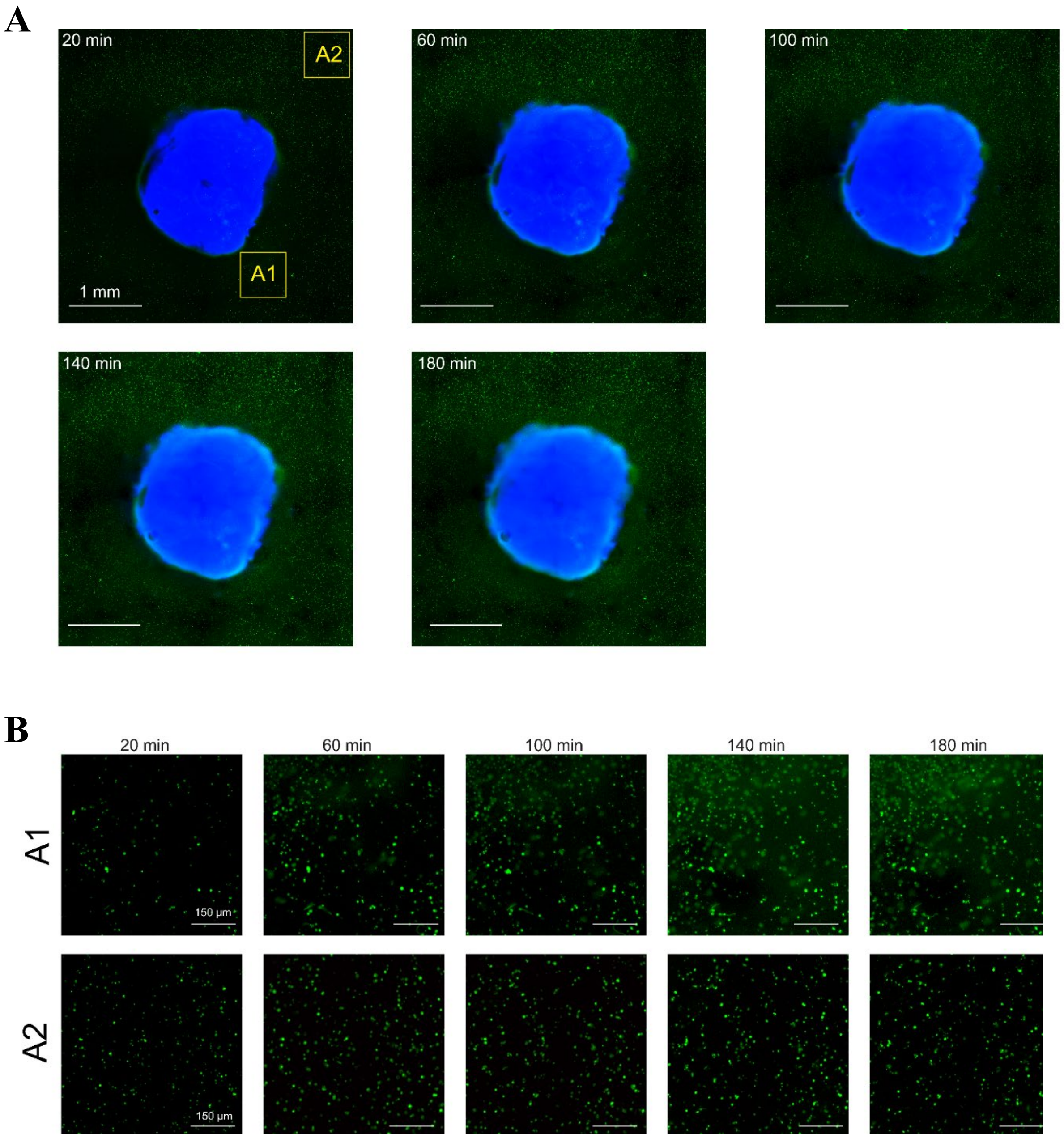

Supplementary Figure 4. Another example of NETosis propagation analysis. The NET score distributions at different time points were acquired from 100 x 100 µm square segments across all regions surrounding a NETs plaque. Graphs were obtained in 40 min intervals for 3 hours.

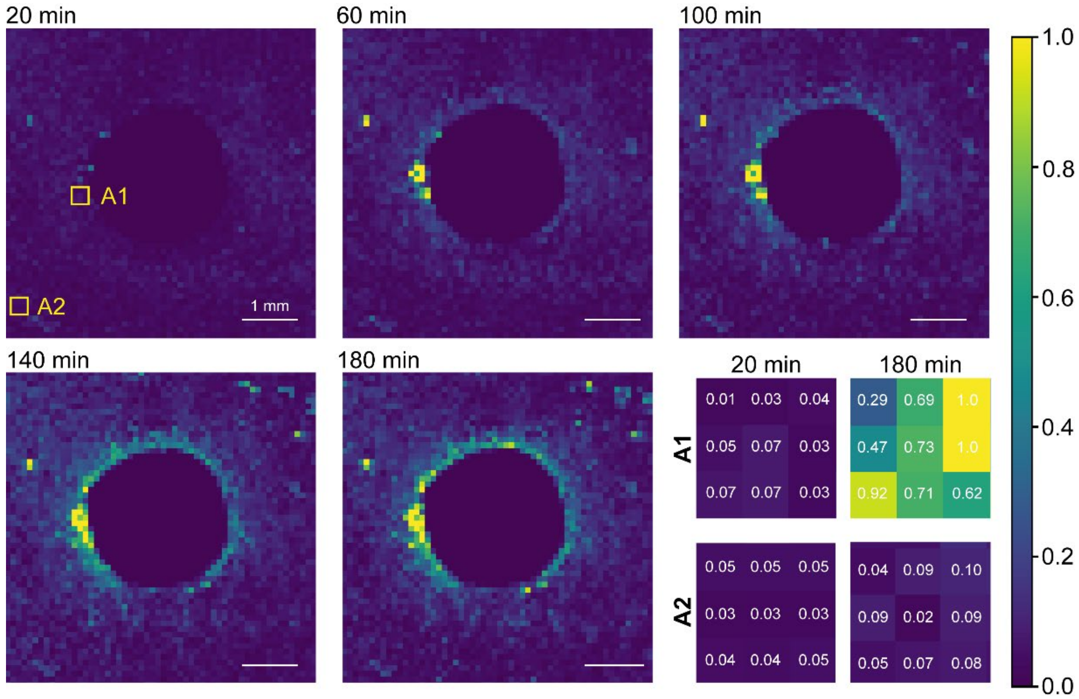

Supplement: Supplementary file 2 — Supplementary Information [file 42003_2024_6074_MOESM2_ESM.pdf]
