# Supplementary material for: Auto-amplification and spatial propagation of neutrophil extracellular traps
Source: Commun Biol. 2024 Mar 29;7:386. doi: 10.1038/s42003-024-06074-z (PMC10980821; doi:10.1038/s42003-024-06074-z)
Supplement: Supplementary file 3 — Description of Additional Supplementary Files [file 42003_2024_6074_MOESM3_ESM.pdf]

Description of Additional Supplementary Files

File name: Supplementary Data 1
Description: Source data for graphs in the main figures.

File name: Supplementary Data 2
Description: Source data for graphs in the Supplementary Figures.
